# Comparison of Two Methods, UHPLC-UV and UHPLC-MS/MS, for the Quantification of Polyphenols in Cider Apple Juices

**DOI:** 10.3390/molecules180910213

**Published:** 2013-08-22

**Authors:** Cindy F. Verdu, Julia Gatto, Ingrid Freuze, Pascal Richomme, François Laurens, David Guilet

**Affiliations:** 1 Laboratoire SONAS, Université d’Angers, SFR QUASAV, Angers 49045, France; E-Mails: cindyverdu@gmail.com (C.F.V.); julia.gatto@univ-angers.fr (J.G.); pascal.richomme@univ-angers.fr (P.R.); 2 Institut de Recherche en Horticulture et Semences, UMR1345, INRA, Université d’Angers, AgroCampus-Ouest, SFR QUASAV, Angers 49045, France; E-Mail: francois.laurens@angers.inra.fr; 3 Plateforme d’Ingénierie et Analyses Moléculaires, Université d’Angers, Angers 49045, France; E-Mail: ingrid.freuze@univ-angers.fr

**Keywords:** *Malus* x *domestica*, LC-UV, LC-MS^n^, phenolic compounds

## Abstract

The aim of this study was to develop faster and more efficient phenotyping methods for in-depth genetic studies on cider apple progeny. The UHPLC chromatographic system was chosen to separate polyphenolic compounds, and quantifications were then simultaneously performed with a UV-PDA detector and an ESI-triple quadrupole mass analyzer (SRM mode). Both quantification methods were validated for 15 major compounds using two apple juice samples, on the basis of linearity, limits of detection and quantification, recovery and precision tests. The comparison between UV and SRM quantifications in 120 different samples of a cider apple progeny showed an excellent correlation for major compounds quantified with both methods. However, an overestimation was revealed for five compounds with the UV detector and the mass analyzer. Co-elution and matrix effects are discussed to explain this phenomenon. SRM methods should therefore be considered with restrictions in some cases for quantification measurements when several phenolic compounds are simultaneously quantified in complex matrices such as apple juices. For both methods, analyses were carried out over short periods of time while maintaining a high quality for the simultaneous quantification of phenolic compounds in apple juice. Each method is relevant for more in-depth genetic studies of the polyphenol content of apple juice.

## 1. Introduction

Cider is essentially produced and consumed in Europe and Canada. Apple varieties and manufacturing processes differ, depending on the country. In France, ciders are the result of a combination of many apple varieties, chosen on the basis of their acidity and tannin content. Cider quality is defined with respect to color, astringency, bitterness, aroma, acidity and sugar content. Some of these traits are directly related to the phenolic content. The polymeric degree of procyanidins influences either the bitterness or the astringency of the beverage, whereas its color is linked to the enzymatic oxidation of phenolic compounds such as procyanidins, (+)-catechin and phloridzin by polyphenol oxidase [1,2]. Some hydroxycinnamic acids may also be the precursors of some volatile compounds responsible for cider aroma [3]. Furthermore, these compounds are widely considered to have a favorable antioxidant potential for human health. Indeed, phenolic compounds have shown *in vitro* anticancer properties [4]. For example, chemoprevention of human colon cancer has been reported for apple procyanidins, whereas it appears that the flavonoids, quercetin and naringenin, lower lung cancer risk [5,6,7]. As far as cardiovascular and coronary heart diseases are concerned, diets high in flavanones and anthocyanidins are associated with a reduced risk of death [8].

Apple, apple juice and cider consumption are inversely correlated with the development of diseases such as asthma, diabetes, cancer and cardiovascular diseases [9,10]. Linked to the *in vitro* effects of phenolic compounds, the favorable effects of apple consumption are often attributed to their high phenolic content related to their high antioxidant potential. Lee and collaborators showed that quercetin glycosides, epicatechin and procyanidin B2 contribute more than vitamin C to the total antioxidant potential of apples [11]. However, it is still not understood how these compounds can transfer their antioxidant potential to the human body and what is the protective role of other constituents such as fibers against these diseases [12,13].

To understand the favorable effects of phenolic compounds on human health, a large number of studies have been devoted to apples in the past. The main classes of polyphenols are monomeric and polymeric flavan-3-ols (e.g., catechins or procyanidins), phenolic acids (e.g., chlorogenic acid), flavonols (e.g., quercitrin) and dihydrochalcones (e.g., phloridzin) [14].

The present study was carried out to develop a rapid, sensitive and reproducible quantification method of phenolic compounds in order to conduct a genetic study based on a large number of progenies. Indeed, despite their great interest for the cider industry, no genetic study had yet been published for cider apples. Our study was based on a cider apple progeny containing 120 different individuals. Because of the high number of samples, a fast phenotyping method was required and the UHPLC method seemed to be the most appropriate. Faster methods have been recently proposed to quantify polyphenols using more powerful chromatographic systems such as UHPLC coupled with a UV detector [15,16] or a mass analyzer [16,17].

Nevertheless, significant differences have already been reported between the quantification of phenolic compounds obtained using the HPLC-UV and HPLC-MS methods. This disparity has often been associated with co-elution phenomena and, more generally, with matrix effects, particularly those observed in HPLC-UV analysis [18].

The aim of this study was to develop, validate and compare two UHPLC methods for quantification of major phenolic compounds of cider apples juices. To define which detector could be adapted to our analytical requirement, two UHPLC methods using a UV-PDA detector and an ESI-triple quadrupole mass analyzer used in Selected Reaction Monitoring (SRM) mode were developed. UHPLC-UV and UHPLC-MS/MS were used simultaneously to quantify 15 major phenolic compounds in cider apple juice. Both methods were separately validated by linearity, limits of detection and quantification, recovery and precision tests. Additionally, both quantifications obtained for 120 samples were used to compare results for major phenolic compounds.

## 2. Results and Discussion

### 2.1. Sample Preparation

During this study, two analytical methods for the quantification of the major polyphenols in apple juices, *i.e.*, UHPLC-UV and UHPLC-MS/MS, were applied to a large number of apple juice samples (a batch of 120 juices in triplicate), leading to long waiting times in the autosampler. To evaluate the stability of phenolic compounds based on former observations, a quantification of each compound was made every 5 h for a storage period (autosampler at 4 °C) of two days. Two sample preparations were tested: the first one consisted in the injection of the raw apple juice, whereas the second one corresponded to a dilution with an equal volume of MeOH with 1% acetic acid. A significantly better stability of compounds over 44 h was obtained under acid conditions (data not shown).

### 2.2. UHPLC-UV & UHPLC-MS/MS Conditions

**Figure 1:**
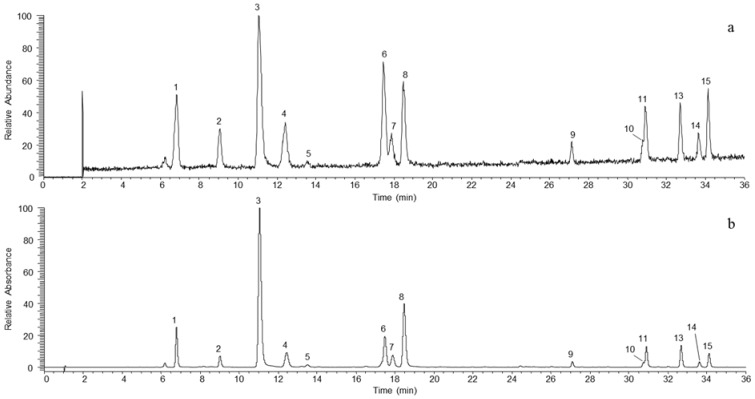
UHPLC-ESI Total Ion Current (TIC, **a**) and UHPLC-UV (λ 280 nm, **b**) chromatograms of standard working solutions. Procyanidin B1 (1), catechin (2), chlorogenic acid (3), procyanidin B2 (4), 4-caffeoylquinic acid (5), epicatechin (6), procyanidin C1 (7), 4-*p*-coumaroylquinic acid (8), procyanidin B5 (9), hyperin (10), phloretin xyloglucoside (11), phloridzin (13), avicularin (14) and quercitrin (15).

**Figure 2:**
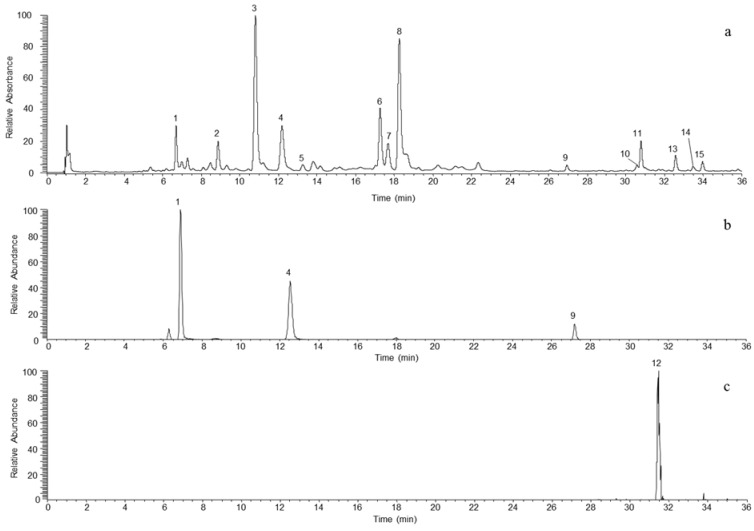
UHPLC chromatograms: (**a**) UV (λ 280 nm); (**b**) SRM for procyanidin; (**c**) SRM for rutin. Procyanidin B1 (1), catechin (2), chlorogenic acid (3), procyanidin B2 (4), 4-caffeoylquinic acid (5), epicatechin (6), procyanidin C1 (7), 4-*p*-coumaroylquinic acid (8), procyanidin B5 (9), hyperin (10), phloretin xyloglucoside (11), rutin (12), phloridzin (13), avicularin (14) and quercitrin (15).

Optimization of the chromatographic conditions was guided by the research of resolution values of adjacent peaks greater than 1.5. Separations were performed with a total run time of 35 min in order to avoid co-elutions that could have significantly impaired the UV quantification of polyphenols (except for compounds **10** and **11**; Figure 1). The analysis time was divided into three segments in the ESI-triple quadrupole mass spectrometer. The first one (0 to 2 min) made it possible to avoid sugar signals. The second one (2 to 28 min) was used to analyze more polar phenolic compounds. In this segment, nine compounds were analyzed in parallel with a scan width and time fixed at 0.5 *m/z* and 0.08 s, respectively. The third segment (28 to 35 min) made it possible to analyze less polar compounds. Six compounds were there analyzed in parallel (0.5 *m/z* and 0.08 s). With these reduced scan times, the digital resolutions were still sufficient for automatic integration and quantification. At the same time, a full scan analyses was completed in the third quadrupole between 150 and 1160 *m/z*, with a scan time of 0.289 s. Additionally, the SRM mode developed in this method allowed an accurate identification with very good signal-to-noise ratios for compounds of interest (Figure 2). Indeed, 4-caffeoylquinic acid, rutin, quercitrin and avicularin compounds could be easily quantified, even when they were barely detected in the TICs of apple juices.

These conditions allowed us to separate major phenolic compounds in 35 min. This time analysis is longer than that reported by Ceyman *et al*. [17]. We chose to increase analysis time to separate all major phenolic compounds present in apple juice, especially to avoid, as much as possible, co-elutions that may affect UV and mass quantifications due to possible matrix effects already reported by other authors [18,19]. The chromatogram obtained under these conditions is available to quantify 25 additional compounds identified primarily as polymeric flavanols, subject to the availability of standards.

### 2.3. Method Validation

Both methods (UHPLC-UV and UHPLC-MS/MS) were validated for quantification of the 15 major apple phenolic compounds, in accordance with performance criteria, by assessing precision, recovery, linearity, LOD and LOQ. The slopes, linear ranges, correlation coefficients of the calibration curves, LOD, LOQ, precision and recovery data are summarized in Table 1 and Table 2. The precision of each method was evaluated including estimations of the intra- and inter-day variations. It is expressed as a relative standard deviation (RSD%). Except for 4-caffeoylquinic acid, intra-day RSD values for all compounds were below 4.0% for the UHPLC-UV method and 5.8% for the UHPLC-MS method. Inter-day RSD varied from 2.6 to 6.2% (11.6% for 4-caffeoylquinic acid) with UV detection, and the values recorded with MS detection were slightly higher with variations ranging from 3.0 to 10.0%. The recovery of the methods was tested in the two apple juices, P12R3A28 and P12R3A67, with the addition of SWS1. For recovery rates, the results obtained ranged from 94.3 to 110.4% with UV detection and from 91.2 to 113.3% with MS detection (Table 1 for P12R3A28 apple juice). The recovery was more than 95% for most of the major phenolic compounds with the two methods. The error range, from 5 to 13%, for epicatechin, procyanidin C1, 4-*p*-coumaroylquinic acid and flavonols, is acceptable, given the number of simultaneous quantified compounds and requirements for genetic studies. The linearity of the method was evaluated with the injection of the SWS at ten injection volumes, in five replicates. For each compound, the range of linearity was assessed after control of the residuals. Calibration data from both methods indicated the linearity for all standards of the UV detection (r^2^ > 0.990) and the MS detection (r^2^ > 0.989). As expected, the LOD and LOQ were higher for UV detection with values—depending on standards—comprised between 0.33 and 4 ng (LOD) and 0.5 and 10 ng (LOQ), when compared to those recorded for MS detection of 0.003 and 2 ng (LOD) and 0.007 and 6.67 ng (LOQ).

**Table 1:** Calibration curve parameters for the 15 major phenolic compounds according to UV detection and results of the validation study for P12R3A28 apple juice.

Compounds ^a^	Regression equation ^b^	Correlation coefficient (r)	Linear range ^c^	LOD ^c^	LOQ ^c^	Juice ^c^	Precision RSD (%)		Added ^c^	Measured ^c^	RSD (%)	Recovery ^f^ (%)
							Intra ^d^	Inter ^e^				
**UHPLC-UV**												
PB1	Y = 21689 + 2153.2X	0.9923	13.30–500	0.3	1.7	24.76	3.3	4.1	10.0	36.26	1.0	95.9
Ca	Y = −489.51 + 3086.7X	0.9989	4.0–150	0.5	2.0	18.63	1.8	4.1	3.0	20.83	2.3	103.8
CA	Y = 45046 + 9890.3X	0.9997	20–1500	<0.5	0.5	110.39	2.6	2.6	30.0	138.14	1.7	101.6
PB2	Y = 13047 + 1674.0X	0.9944	13.3–500	0.7	6.7	76.82	2.6	2.6	10.0	92.1	2.7	94.3
4CA	Y = 8413 + 8554.6X	0.9912	1.67–25	0.3	1.7	2.44	5.3	11.6	0.5	3.00	9.5	98.0
ECa	Y = −23692 + 3783.4X	0.9971	33.3–500	0.7	1.7	67.18	3.4	2.9	10.0	73.37	1.1	105.2
PC1	Y = 12620 + 1922.0X	0.9900	20–300	1.0	4.0	51.58	3.7	3.2	10.0	57.29	5.8	107.5
4PCQA	Y = 9224.5 + 10112X	0.9999	1.67–500	0.3	0.7	22.16	2.2	2.6	10.0	32.23	2.2	99.8
PB5	Y = −390.79 + 3058.7X	0.9995	1.78–66.5	0.9	1.8	4.51	4.0	4.2	1.3	5.74	2.1	101.7
QGa	Y = −1292.4 + 7250.6X	0.9994	1.33–50	3.3	10.0	5.18	1.3	2.9	1.0	5.78	4.7	106.9
PLXG	Y = 935.15 + 5226.0X	0.9997	2.0–150	0.5	1.0	18.65	2.1	2.8	3.0	22.19	4.5	97.6
QR	nd	nd	nd	nd	nd	nd	nd	nd	0.1	nd	nd	nd
PLG	Y = −782.23 + 8112.4X	0.9998	1.33–100	0.3	1.3	6.86	1.8	3.2	2.0	8.54	2.1	103.8
QA	Y = −665.42 + 7232.9X	0.9991	3.33–50	3.3	10.0	3.35	2.2	3.8	1.0	3.94	3.0	110.4
QRh	Y = −2177.8 + 5159.0X	0.9994	4.0–150	4.0	10.0	3.32	1.7	6.2	3.0	6.04	1.4	104.6

^a^ PB1: procyanidin B1; Ca: (+)-catechin; CA: chlorogenic acid; PB2: procyanidin B2; 4CA: 4-caffeoylquinic acid; ECa: (−)-epicatechin; PC1: procyanidin C1; 4PCQA: 4-*p*-coumaroylquinic acid; PB5: procyanidin B5; QGa: hyperin; PLXG: phloretin xyloglucoside; QR: rutin; PLG: phloridzin; QA: avicularin; QRh: quercitrin. ^b^ y = peak area; x = concentration of compound (µg mL^−1^). ^c^ in nanograms. ^d^ intra-day (n = 5). ^e^ inter-day (n = 3 × 3). ^f^ recovery (%) = (amount_juice_ + amount_added_)/amount_measured_ x 100. nd: not detected.

**Table 2:** Calibration curve parameters for the 15 major phenolic compounds according to MS detection and results of the validation study for P12R3A28 apple juice.

Compounds ^a^	Regression equation ^b^	Correlation coefficient (r)	Linear range ^c^	LOD ^c^	LOQ ^c^	Juice ^c^	Precision RSD (%)		Added ^c^	Measured ^c^	RSD (%)	Recovery ^f^ (%)
							Intra ^d^	Inter ^e^				
**UHPLC-MS/MS**												
PB1	Y = −42711 + 39800X	0.9970	6.67–500	0.2	0.17	23.89	2.6	10.7	10.0	34.14	2.7	99.3
Ca	Y = −92586 + 46791X	0.9938	4.0–150	2.0	4.0	15.31	4.4	9.8	3.0	18.44	5.7	99.3
CA	Y = 7634200 + 302370X	0.9922	20–1500	0.5	1.0	126.16	3.2	6.8	30.0	171.18	2.9	91.2
PB2	Y = −3107.7 + 29137X	0.9968	1.67–500	0.2	0.33	66.06	3.7	8.1	10.0	77.18	3.1	98.6
4CA	Y = 39949 + 297480X	0.994	0.33–25	0.08	0.33	1.99	6.8	9.0	0.5	2.42	2.8	102.7
ECa	Y = −328880 + 53294X	0.9957	13.3–500	1.7	6.7	69.88	5.1	8.8	10.0	80.03	3.7	99.8
PC1	Y = 12323 + 20204X	0.9976	4.0–300	<0.1	0.2	34.01	0.3	4.9	10.0	41.00	1.4	107.4
4PCQA	Y = −1655300 + 131390X	0.9893	0.67–500	0.17	0.67	21.46	2.5	3.0	10.0	27.76	2.6	113.3
PB5	Y = −9772.9 + 37935X	0.9961	0.89–66.5	0.08	0.2	3.24	2.3	5.07	1.3	4.46	4.9	102.4
QGa	Y = −66428 + 333280X	0.9977	0.67–50	<0.02	0.02	4.9	2.8	3.5	1.0	5.76	3.8	102.4
PLXG	Y = 565330 + 480430X	0.9981	2.0–150	<0.05	1.0	21.46	3.9	5.0	3.0	24.81	3.0	98.6
QR	Y = −10222 + 570740X	0.9991	0.070–5	0.003	0.007	0.1	7.2	3.7	0.1	0.19	5.7	102.8
PLG	Y = −4619.6 + 198410X	0.997	0.33–100	<0.03	0.03	6.68	5.4	5.2	2.0	8.33	3.1	104.1
QA	Y = −61100 + 84940X	0.9973	1.33–50	0.17	1.3	2.84	5.8	4.0	1.0	3.46	2.7	110.9
QRh	Y = −218580 + 174400X	0.9972	2.0–150	<0.05	0.05	3.68	4.2	3.9	3.0	5.96	1.4	112.1

^a^ PB1: procyanidin B1; Ca: (+)-catechin; CA: chlorogenic acid; PB2: procyanidin B2; 4CA: 4-caffeoylquinic acid; ECa: (−)-epicatechin; PC1: procyanidin C1; 4PCQA: 4-*p*-coumaroylquinic acid; PB5: procyanidin B5; QGa: hyperin; PLXG: phloretin xyloglucoside; QR: rutin; PLG: phloridzin; QA: avicularin; QRh: quercitrin. ^b^ y = peak area; x = concentration of compound (µg mL^−1^). ^c^ in nanograms. ^d^ intra-day (n = 5). ^e^ inter-day (n = 3 × 3). ^f^ recovery (%) = (amount_juice_ + amount_added_)/amount_measured_ × 100.

### 2.4. Comparison of the UHPLC-UV & UHPLC-MS/MS Methods

Apple juices were prepared from 120 progenies and were analyzed in triplicate in both methods. Table 3 shows the range of each compound present in the apple juices prepared from the apple progeny studied. The major compound of apple juice is chlorogenic acid, with concentrations ranging from 97.23 to 741.1 µg/mL apple juice. Procyanidin B2 is the second major compound with concentrations ranging from 76.2 to 355.7 µg/mL. The least concentrated compound is rutin, with a concentration ranging from 0.16 to 1.75 µg/mL. These results are in accordance with former studies published on cider apple juice [20].

**Table 3:** Range of concentrations of different phenolic compounds quantified with the UV-PDA detector and the ESI-triple quadrupole mass analyzer in 120 apple juices prepared from the progeny, X5210 × X8402.

Compounds ^a^	UHPLC-UV		UHPLC-MS/MS	
	Min ^b^	Max ^b^	Min ^b^	Max ^b^
PB1	16.7	231.7	39.1	326.7
Ca	5.6	138.2	11.9	105.5
CA	89.3	2240.9	97.3	741.1
PB2	120.1	650.3	nq	nq
4CA	3.4	17.8	1.8	12.4
ECa	54.3	298.5	49.4	246.8
PC1	54.9	269.7	54.1	242.8
4PCQA	2.9	394.1	12.2	126.3
PB5	6	35.7	nq	nq
QGa	6.7	23.7	2.3	12.0
PLXG	13.3	113.9	14.6	126.9
QR	nd	nd	0.2	1.7
PLG	7.5	74.1	6.3	44.3
QA	6.8	20.6	2.8	10.3
QRh	9.2	76.6	3.8	37.2

^a^ PB1: procyanidin B1; Ca: (+)-catechin; CA: chlorogenic acid; PB2: procyanidin B2; 4CA: 4-caffeoylquinic acid; ECa: (−)-epicatechin; PC1: procyanidin C1; 4PCQA: 4-*p*-coumaroylquinic acid; PB5: procyanidin B5; QGa: hyperin; PLXG: phloretin xyloglucoside; QR: rutin; PLG: phloridzin; QA: avicularin; QRh: quercitrin. ^b^ in µg mL^−1^ apple juice. nd: not detected. nq: not quantified.

**Figure 3:**
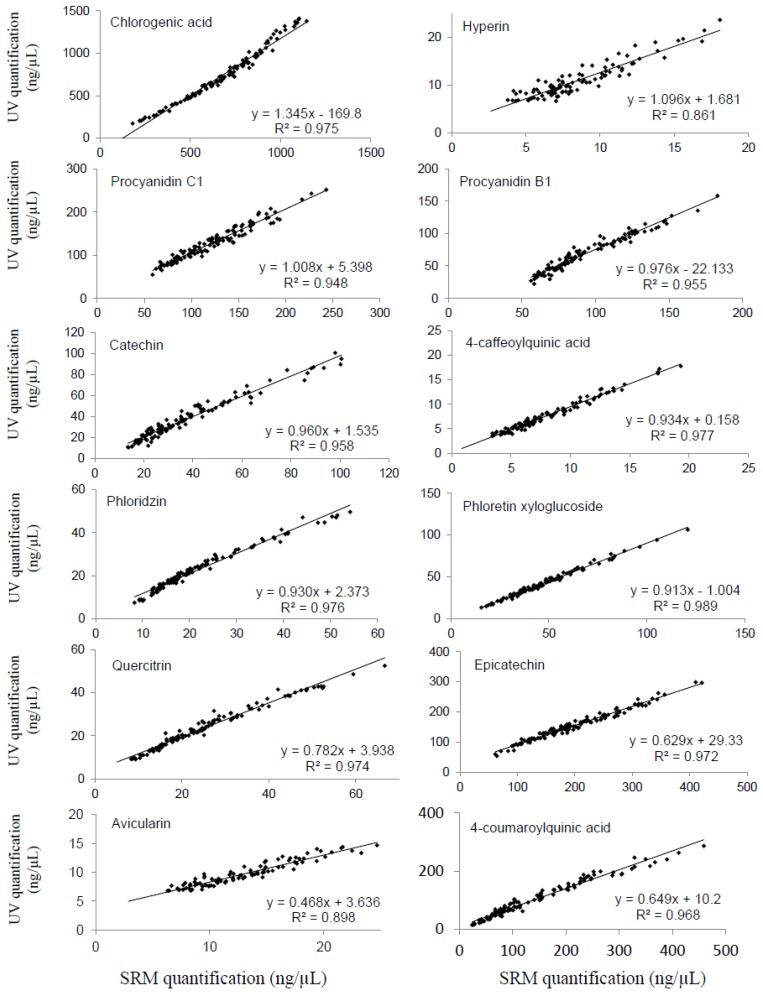
Comparison of the quantifications of 120 apple juices (n = 3, P value < 0.0001) with the UV- PDA detector (X-axis) and the ESI-triple quadrupole mass analyzer (Y-axis).

For all standards, the concentrations measured by the two methods in apple juices were in agreement over the range of calibration, with correlation coefficients r^2^ > 0.948, except for avicularin and hyperin, with r^2^ = 0.898 and r^2^ = 0.861, respectively (Figure 3). The slopes of the linear regression obtained by comparison of the quantification of 120 apple juices with the UV-PDA detector and the ESI-triple quadrupole mass spectrometer (Figure 3) ranged from 0.468 (avicularin) to 1.345 (chlorogenic acid). For hyperin, procyanidin B1, procyanidin C1, catechin, 4-caffeoylquinic acid, phloridzin and phloretin xyloglucoside, the slope values (around 1.0 ± 0.1) indicated that UV quantification was in accordance with MS/MS quantification.

There seems to be no systematic bias for these compounds in both methods (no co-elution phenomena, no matrix effect). Despite the independent validation of the two methods, the quantifications were not equivalent for five compounds. On one hand, chlorogenic acid was overestimated with the UV-PDA detector compared to the MS quantification (slope value: 1.345). Matrix effects are generally associated with response suppression effects, as observed in ESI-MS quantification, when compared to other detection methods [18,21]. Under our conditions, a co-elution phenomenon probably explains the UV/MS differences in chlorogenic acid estimations [22]. Indeed, this analyte was co-eluted with an unidentified compound with positive UV absorbance at 320 nm and which was not quantified under SRM conditions with MS filters at 353 and 192 uma. This hypothesis was supported by the emergence of a weak peak at 436.7 uma under UHPLC-MS conditions, at the same retention time as chlorogenic acid. Despite the use of the UHPLC system and a relatively long time analysis, the chromatographic resolution was not sufficient to avoid co-elutions for concentrated compounds such as chlorogenic acid in apple juices. The use of SRM was therefore well adapted in this case.

On the other hand, quercitrin, epicatechin and 4-*p*-coumaroylquinic acid, as well as avicularin, were underrated with the UV-PDA detector compared to the MS quantification (slope values: 0.782, 0.629, 0.649 and 0.468, respectively). Several studies have already reported similar results without any additional explanation [18,19]. Since we compared the results obtained from simultaneous UV and MS analysis here, samples or standard preparations could not be implicated in these variations. Results could be explained by matrix effects, often highlighted in LC-ESI-MS, with an increasing ionization response associated with co-eluting components. The recovery test results obtained for the P12R3A28 apple juice for these compounds seem to be in accordance with this hypothesis since four of the five results obtained are the highest ones in this assay. However, results obtained for the second apple juice, P12R3A67, were better and did not show any matrix effects. Nevertheless, it remains difficult to draw conclusions on the basis of these results. It therefore appears that LC-MS quantifications cannot be ubiquitously applied to the linear quantification of all phenolic compounds, especially when dealing with complex matrixes such as apple juices. Further studies will then be required to validate our explanations. Hernando and collaborators have already proposed to greatly dilute the sample in order to reduce the presence of matrix interference [23]. However, this is only applicable for some apple juices that are sufficiently concentrated to be quantified with both methods.

## 3. Experimental

### 3.1. Standards and Chemicals

LC/MS-grade MeOH was purchased from Carlo Erba Reagents (Val de Reuil, France). Formic acid and acetic acid of LC/MS grade were obtained from Fisher Scientific (Illkirch, France). Ultrapure water was obtained from a MilliQ water purification system (Millipore S.A., Molsheim, France). Standards of procyanidins B1, B2, B5 and C1, 4-*p*-coumaroylquinic acid, 4-caffeoylquinic acid and phloretin xyloglucoside were obtained from Polyphenol Biotech (Bordeaux, France). (+)-Catechin, (−)-epicatechin, chlorogenic acid, phloridzin and rutin were purchased from Sigma-Aldrich (Lyon, France). Hyperin was obtained from Extrasynthese (Genay, France), avicularin was obtained from LGC Standards SARL (Molsheim, France), and quercitrin was purified in the laboratory (more than 80% purity).

### 3.2. UHPLC-UV-MS Instrumentation and Conditions

All UHPLC analyses were performed using a Thermo Accela High Speed LC system (Thermo Scientific, Gometz le Châtel, France) equipped with a refrigerated autosampler. Samples were injected into a Zorbax Eclips Plus C18 column (2.1 × 50 mm, 1.8 µm; Agilent) using a 10-µL loop in partial loop mode. The column was heated at 30 °C and was equipped with an in-line filter (0.2 µm) (Thermo Scientific). The following solvents were used: (A) 0.1% formic acid in water, and (B) methanol with a time gradient mode T (min)/%B: 0/10, 1/10, 3/18, 11/18.5, 13/21.5, 17/25.5, 21/29, 23/32, 35/50. The flow rate was set at 250 µL/min (500 bars).

The UV experiments were performed with a Thermo Accela PDA detector. Hydroxycinnamic acids were detected at 320 nm and dihydrochalcones, flavonols and flavanols were detected at 280 nm.

The MS experiments were performed with a Thermo TSQ Quantum Access MAX equipped with an electrospray interface (ESI) operating in the negative ionization mode. Each standard was infused into the electrospray ion source at 5 µg/mL in MeOH using a syringe pump at a flow rate of 250 μL/min to determine the collision energy, the tube lens offset and the SRM transitions chosen to be the most sensitive with the lowest collision energy for each compound (Table 4).

**Table 4:** Retention time (Rt), MS/MS fragment ions, collision energy (CE) and Tube Lens Offset (TLO) for the 15 major phenolic compounds of apple juices.

Compounds ^a^	R_t_ (min)	Precursor ion [M−H]^−^ (*m/z*)	Fragments [M−H]^−^ (*m/z*)	CE (V)	TLO (a.u.)
PB1	6.8	577	289	30	159
Ca	9.1	289	245	16	159
CA	11.2	353	191	22	62
PB2	12.5	577	289	30	159
4CA	13.5	353	173	20	58
ECa	17.6	289	245	16	159
PC1	18.0	865	289	45	159
4PCQA	18.6	337	173	16	159
PB5	27.2	577	289	30	159
QGa	30.8	463	300	32	159
PLXG	31.0	567	273	20	139
QR	31.6	609	300	40	159
PLG	32.8	435	167	32	159
QA	33.7	433	300	15	160
QRh	34.2	447	300	32	159

^a^ PB1: procyanidin B1; Ca: (+)-catechin; CA: chlorogenic acid; PB2: procyanidin B2; 4CA: 4-caffeoylquinic acid; ECa: (−)-epicatechin; PC1: procyanidin C1; 4PCQA: 4-*p*-coumaroylquinic acid; PB5: procyanidin B5; QGa: hyperin; PLXG: phloretin xyloglucoside; QR: rutin; PLG: phloridzin; QA: avicularin; QRh: quercitrin.

The Selected Reaction Monitoring (SRM) mode was used to quantify phenolic compounds. The ESI conditions were as follow: spray voltage, 3500 V; vaporizer temperature, 350 °C; sheath gas pressure, 48 arbitrary units (au); ion sweep gas, 1 au; auxiliary gas pressure, 13 au; capillary temperature, 200 °C; skimmer offset, 0 au. The collision gas used was argon at a pressure of 1.5 mTorr. The data were processed using Xcalibur software (2.1). The retention times (Rt) of each compound are also listed in Table 4.

Moreover, the method described made it possible to separate and quantify the major phenolic compounds of apple juices in 35 min. Quantifications were simultaneously performed with a UV-PDA detector and an ESI-triple quadrupole mass spectrometer. The spectrometer divert valve was set to the waste position during the first minute to prevent more polar compounds such as polysaccharides from entering the ion source.

### 3.3. Preparation of Standard Solutions

A 1 mg/mL stock solution was prepared for each standard in MeOH and stored at −80 °C (except for chlorogenic acid and epicatechin: 10 mg/mL). Stock solutions were combined into one single solution according to the expected relative proportions of each compound in targeted apple juices. This single solution was further diluted in order to prepare different working solutions (SWS1, SWS2 and SWS3; data not shown). Calibration curves were fitted for each standard using ten different final concentrations corresponding to the appropriate range for each compound (e.g., 0.5–500 ng injected for catechin; 0.015–50 ng injected for hyperin). Five replicates were taken and the mean linear regression of the mass of analyte injected versus its peak area was used as a calibration curve. The SWS solutions were stored at −80 °C.

### 3.4. Sample Preparation

The progeny used in this study was derived from two INRA hybrids, X5210 and X8402, crossed in 2000, and composed of 120 trees. The former (X5210) is derived from the cider variety “Kermerrien”, whereas the latter (X8402) is a dessert apple hybrid whose grandparents include the two varieties, “Florina” and “Prima”. Trees were planted on their own roots in 2003 in the orchards of the Horticulture Experimental Unit at INRA, Angers-Nantes.

Fruits (1 kg/tree) were harvested between September and November 2010 at the mature stage “50% of fallen fruits” which is the harvest stage in commercial cider orchards. The two hybrids used to validate the method (P12R3A28 and P12R3A67) were harvested in September 2009. Whole fruits were then cored and crushed to extract the juices. Sodium fluoride was added to stop phenolic oxidation and apple juices were stored at −80 °C.

Sample stability was estimated with three replicates per tree prepared according to the two methods described above and stored at −80 °C. Samples were then defrosted, mixed and placed in the autosampler 5 min before analysis began. Full analysis was completed after 44 h with injections every 5 h. Stability was estimated through ANOVA analysis.

### 3.5. Validation Study

The method was developed on the 15 major phenolic compounds present in apple juices. Limits of detection (LOD) and quantification (LOQ), linearity, recovery and precision of the method were evaluated.

For the LC-UV and LC-MS/MS methods, LOD and LOQ were estimated by injecting serial dilutions of working solutions with the final criterion signal-to-noise ratio (S/N) of 3 and 10, respectively. The linearity of the calibration curves was assessed by injecting ten volumes of SWS in five replicates. Residuals (difference between nominal concentration and calculated concentration by the linear model) and their distribution (normally distributed around the mean) were monitored. The recovery of the method was tested in both apple juices, P12R3A28 and P12R3A67, with the addition of 100 µL of SWS1. These two mixes (P12R3A28-SWS1 and P12R3A67-SWS1) were analyzed in triplicate (1 µL injection). Results were expressed for each analyte by comparing their levels in spiked samples with those obtained in initial juices to which a known amount of analyte was added: recovery (%) = (amount_juice_ + amount_added_)/amount_measured_ × 100. The precision of the method, expressed by the relative standard deviation (RSD%), was estimated by measuring the compound levels in several replications of both apple juices. The intra-day variation was evaluated on five replicates of both apple juices, whereas the inter-day variation was evaluated on three replicates per juice on three different days.

## 4. Conclusions

The use of the UHPLC system to separate phenolic compounds in apple juice allowed a more sensitive and more rapid analysis than conventional HPLC. Both methods developed in this study for the quantification of phenolic compounds in apple juices by UHPLC-UV and UHPLC-MS/MS were separately validated on the basis of LOD, LOQ, linearity, recovery and precision tests. A total of 120 different samples of cider apple juice were analyzed using these two methods. Comparison of the quantifications of the 12 major compounds in the cider apple juices with the UV detector and the ESI-triple quadrupole mass analyzer showed good correlations for all compounds, ranging between 0.860 and 0.989. However, the slope value showed an overestimation of the UV detector for chlorogenic acid and an overestimation of the mass analyzer for epicatechin, 4-*p*-coumaroylquinic acid, avicularin and quercitrin. The overestimation with the UV-PDA detector could be explained by the co-elution of chlorogenic acid with an unknown UV-absorbing minor compound, highlighting the advantage of using the SRM mode to quantify highly concentrated compounds. For the four other compounds, some matrix effects may be responsible of the overestimation, despite the use of high resolutive chromatographic technology as UHPLC.

Even if these five compounds are relatively overestimated with one detector, the high correlation coefficient obtained indicates that both methods are well adapted for genetic studies. Moreover, UHPLC-based methods make it possible to significantly reduce the analysis time and to provide a better resolution, compared to HPLC methods. Given the large number of samples required for genetic analysis, UHPLC is the preferable method to be used in genetic studies.
